# T cell immunophenotypes and IgE responses in patients with moderate‐to‐severe atopic dermatitis receiving dupilumab

**DOI:** 10.1002/clt2.70062

**Published:** 2025-05-08

**Authors:** Davender Redhu, Wojciech Francuzik, Philipp Globig, Margitta Worm

**Affiliations:** ^1^ Division of Allergy and Immunology Department of Dermatology, Venerology and Allergy Charité Universitätsmedizin Berlin Corporate Member of Freie Universität Berlin and Humboldt‐Universität zu Berlin Berlin Germany

**Keywords:** atopic dermatitis, dupilumab, IgE, immune modulation, T cell phenotyping

## Abstract

**Background:**

Targeting the interleukin‐4 receptor alpha (IL‐4Rα) subunit has proven clinical efficacy in atopic dermatitis (AD).

**Objective:**

This study assessed the peripheral phenotype and function of T‐cells, but also levels of total and sIgE and its receptors in AD patients receiving dupilumab.

**Methods:**

AD patients were clinically assessed (*n* = 75) and peripheral blood samples were taken (*n* = 25). Multiparametric flow cytometry was performed to characterize T‐cell subsets (before treatment and 6 months later). Total and specific IgE were measured by ImmunoCap, soluble CD23 and FcεR1 in serum by ELISA, and eosinophils by differential blood analysis.

**Results:**

SCORing Atopic Dermatitis scores and body surface area involvement decreased upon treatment after 6 months of treatment to 67% and 77% from baseline. At the T cell level, we observed a 0.55‐fold reduction of Th2‐cells and a mean 27% increase in regulatory T‐cells from baseline, accompanied by shifts towards Th1 and Th17 phenotypes. Furthermore, circulating CD4^+^CXCR5^+^TFH17 and CD4^+^CXCR5^+^TFH17.1 positive cells (mean 40% and 42%) and T‐cell‐specific IL‐2 (+0.96‐fold) and IL‐10 (+1.96‐fold) secretion increased, whereas IL‐4 (mean −55%) and IL‐17A (mean −27%) were reduced. Eosinophil counts (mean −22%), total IgE (mean −47%) and House Dust Mite sIgE (mean −40%) decreased, whereas CD23 and FcεR1 remained unchanged.

**Conclusions:**

The T‐cell and cytokine profiles during anti‐IL4‐Ra treatment suggest that targeting this pathway promotes a systemic shift of the T‐cell compartment by reducing the T helper type 2 and complementary IgE responses. The sustainability of these disease‐modifying effects requires further investigation.

## 1 INTRODUCTION

Atopic dermatitis (AD) is a chronic inflammatory skin disease characterized by eczematous lesions, pruritus, and xerosis.^1^ The pathogenesis of AD involves a complex interplay of genetic, environmental, and immunological factors, with dysregulation of T helper type 2 (Th2) immune responses playing a pivotal role.^2^, ^3^

Dupilumab, a monoclonal antibody targeting the interleukin‐4 receptor alpha (IL‐4Rα) subunit, has emerged as a promising therapeutic option for moderate‐to‐severe AD.^4^, ^5^, ^6^, ^7^ By inhibiting IL‐4 and IL‐13 signaling, dupilumab reduces Th2‐driven inflammation, leading to significant improvements in clinical outcome.^3^, ^8^, ^9^ While the clinical efficacy of dupilumab has been well‐established, its impact on specific T cell subsets and cytokine profiles in AD patients remains incompletely understood.^10^

To date, several reports suggest that dupilumab may promote immune shifts associated with an increased risk of Th1/Th17‐mediated autoimmune diseases. These include the onset of psoriasis,^11^, ^12^, ^13^ inflammatory arthritis,^14^ alopecia areata^15^, ^16^, ^17^ and rosacea.^18^ A suppression of Th2 responses and a subsequent polarization of the T cell repertoire towards pro‐inflammatory Th1 and Th17 pathways^17^ has been suggested as a possible mechanism.

In this study, we analyzed the phenotype and function of peripheral T cells, but also their consecutive systemic IgE response in AD patients receiving anti‐IL‐4Rα targeting over 6 months.

## 2 MATERIAL AND METHODS

### 2.1 Study design and clinical data collection

Twenty‐five adult patients (18–75 years) with moderate‐to‐severe AD, confirmed for at least 1 year and with at least 10% body surface area (BSA) involvement (IGA score ≥3) (*n* = 25), were enrolled in this study after obtaining informed consent (EA1/122/19). Patients not included if they had concurrent skin conditions, a recent use of other biologics or investigational drugs, or any history of a rheumatic disease. Participants received a 600 mg loading dose of dupilumab (commercially available as *Dupixent®*, manufactured by Sanofi and Regeneron Pharmaceuticals) followed by 300 mg every 2 weeks. AD severity was assessed using patient‐reported outcomes (pruritus, sleep loss) and investigator‐assessed measures, SCORing Atopic Dermatitis (SCORAD) and BSA (*n* = 75).^19^, ^20^ Due to logistical and ethical limitations, the number of patients with clinical scores (*n* = 75) was not identical with those who underwent immunophenotyping (*n* = 25) as peripheral blood sampling for immunological analysis required additional patient consent which was declined by some patients.

## 3 ELISA

Total and specific IgE measurement on serum samples was performed using the ImmunoCap test (Thermo‐Fisher). Soluble CD23 (sCD23) and soluble FcεRI were measured as surrogate markers of Th2‐mediated immune activation in AD.^21^ sCD23 (Quantikine ELISA Kit, R&D Systems) and soluble FcεR1 (ELISA Kit, Invitrogen) in serum were quantified by ELISA as per manufacturer's instructions.

### 3.1 PBMCs isolation

PBMCs were isolated by density gradient centrifugation using Ficoll‐Paque (GE Healthcare). Subsequently, the isolated PBMCs were cryopreserved in Recovery‐Cell Culture Freezing Medium (Gibco) at a density of 20 × 10^6^ cells/mL and transferred into cryovials and stored at −170°C until further use.

### 3.2 Flow cytometry

Thawing of PBMCs was carried out in a 37°C water bath, followed by washing and resuspension in Rosewell Park Memorial Institute 1640 medium (Gibco) supplemented with 10% fetal bovine serum (FBS, Bio&Sell, Lot# BS.338199D) and 1% penicillin‐streptomycin (Gibco, Lot# 2087433).

A total of 1 × 10^6^ PBMCs were plated in a round bottom 96‐well plate overnight, followed by stimulation with phorbol 12‐myristate 13‐acetate (25 ng/mL, Sigma‐Aldrich)‐Ionomycin (2 µg/mL, Sigma‐Aldrich) in presence of Brefeldin A (10 μg/mL, Sigma‐Aldrich) for 4 h. To determine cell death, Live/DEAD Fixable Near‐IR dead cell dye (Invitrogen) was used. Surface staining of multiple T‐cell markers (see Table S1) was performed for 15 min at room temperature using Fluorescence‐Activated Cell Sorting (FACS) buffer (0.1% bovine serum albumin, Serva electrophoresis GmbH, Lot# 2087433, in Phosphate‐Buffered Saline).

For intracellular cytokine production, cells were fixed and permeabilized by using eBioscience fixation and permeabilization buffers (Invitrogen) and stained for 30 min using FACS buffer for cytokines as specified in Table S2. Data were acquired on a CytoFLEX flow cytometer (Beckman Coulter Gmbh) and analyzed by FlowJo software.

### 3.3 Statistical analysis

Statistical analyses were performed using Prism (version 9, GraphPad Software). Data are presented with mean ± SD. The Wilcoxon signed rank paired test was used to compare two continuous variables in the same patient. *p* ≤ 0.05 were considered statistically significant.

## 4 RESULTS

### 4.1 Real‐world clinical outcomes

**FIGURE 1:**
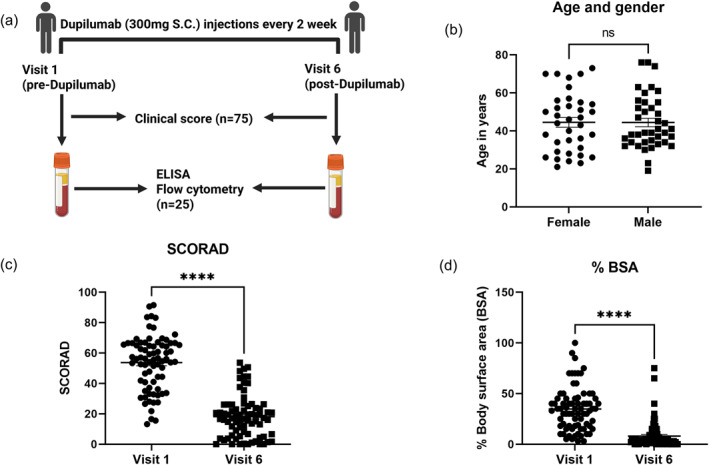
Clinical response following dupilumab treatment. (A) Graphical depiction of the study design. (B) Age (18–75 years) and gender distribution (36 female, 39 male) of the study population. (C) SCORAD. (D) Percent body surface area before and after dupilumab treatment, demonstrating significant clinical improvement. SCORAD, SCORing Atopic Dermatitis. Statistical significance: ^∗∗∗∗^ *p* < 0.0005.

Figure 1A,B show the study design, the sex distribution of the recruited cohort and their clinical response. The SCORAD (Figure 1C, 66% reduction from mean baseline, ^∗∗∗∗^ *p* < 0.0005) and percent BSA (Figure 1D, 77% decrease from mean baseline, ^∗∗∗∗^ *p* < 0.0005) significantly improved after 6 months of treatment in this real‐life setting.

### 4.2 Phenotype of memory T cell subsets

**FIGURE 2:**
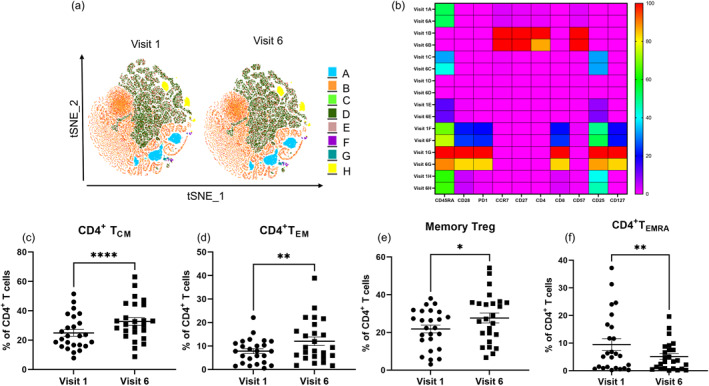
Identification and modulation of memory T cell subsets upon anti‐IL‐4Rα treatment. (A) tSNE plot showing eight distinct cell clusters using FlowSOM. (B) Cumulative heatmap analysis encompassing all patients and timepoints (*n* = 25), presenting the relative expression levels of markers (CD45RA, CD28, PD‐1, CCR7, CD27, CD4, CD8, CD57, CD25 and CD127). Each row within the heatmap corresponds to a single cluster and each column represents one marker. Color intensity reflects expression level (red: high, blue: low). (C) Percentages of CD4^+^ T_CM_ (CD45RA^−^CCR7^+^) (D) T_EM_ (CD45RA^−^CCR7^−^) (E) Memory Treg (CD45RA^−^CD127^−^) and (F) T_EMRA_ (CD45RA^+^CCR7^−^) T cells in PBMCs from patients with AD before and after 6 months of dupilumab treatment. Data are presented as mean ± SD (*n* = 25). Statistical significance: ^∗^ *p* < 0.05, ^∗∗^ *p* < 0.005, ^∗∗∗∗^ *p* < 0.0005. T_CM_, T central memory, T_EM_, T effector memory and T_EMRA_, RA^+^effector memory T cells, Treg, Regulatory T cells.

First, we investigated the peripheral memory T cell subtypes by applying multiparametric surface staining (CD45RA, CD28, PD‐1, CCR7, CD4, CD8, CD57, CD25 and CD127). The tSNE and FlowSOM algorithm plugins were used to cluster and analyze the T cell subsets before and during anti‐IL‐4Rα treatment (Figure 2A).

We identified distinct expression patterns among the T cell populations and clustered them according to their phenotypic properties (A–H, see Figure 2B). Upon treatment, we observed a significant increase in clusters representing central memory T cells (TCM) (cluster B, CD4^+^ CD45RA^−^ CCR7^+^), effector memory T cells (TEM) (cluster C, CD4^+^ CD45RA^−^ CCR7^−^), memory regulatory T cells (memory Treg) (cluster H) and a decrease in terminally differentiated effector memory T cells re‐expressing CD45RA (TEMRA)‐like cell phenotype (cluster G, CD4^+^ CD45RA^+^ CCR7^−^), while rest of the clusters remained stable.

The expression patterns of various markers were validated using biaxial gating to identify TCM (CD4^+^ CD45RA^−^ CCR7^+^), TEM (CD4^+^ CD45RA^−^ CCR7^−^), memory Treg (CD4^+^ CD45RA^−^ CD127^−^) and TEMRA (CD4^+^ CD45RA^+^ CCR7^−^) T cell subtypes before and after dupilumab treatment. Notably, the frequency of circulating TCM cells significantly increased following treatment, showing a mean increase of 31% from baseline (****p < 0.0005) (Figure 2C). TEM cells exhibited a significant increase (mean 54% from baseline, **p < 0.01) after treatment (Figure 2D). Additionally, we observed an enhanced frequency of circulating memory Treg cells (mean −27% from baseline, ^∗^ *p* < 0.05) during dupilumab treatment (Figure 2E). In contrast to TCM, TEM and memory Treg, the percentage of TEMRA cells decreased significantly, with a mean reduction of 46% from baseline (Figure 2E). No differences were observed in the CD8^+^ memory T cell compartment between the treated and untreated groups in the PBMCs derived from AD patients (data not shown).

### 4.3 Phenotype of circulating TFH cell subsets

**FIGURE 3:**
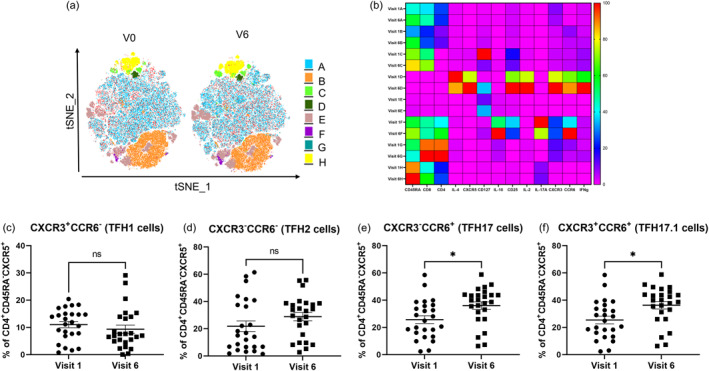
Identification and shift of TFH cell subsets following anti‐IL‐4Rα treatment. (A) tSNE plot, illustrating eight distinct CD3^+^ T cell clusters (A–H) identified via FlowSOM analysis in patients with AD before (Visit 1) and after 6 months (Visit 6) of anti‐IL‐4Rα treatment. (B) Cumulative heatmap analysis encompassing all patients and timepoints (*n* = 25), presenting the relative expression levels of markers (CD45RA, CD8, CD4, IL‐4, CXCR5, IL‐10, CD25, IL‐2, IL‐17A, CXCR3, CCR6 and IFNg) across the eight CD3^+^ cell clusters at Visit 1 and Visit 6. Each row within the heatmap corresponds to a single cluster and each column represents one marker. Color intensity reflects expression level (red: high, blue: low). (c–f) Frequencies of specific TFH subsets before and after treatment: (C) TFH1 (CD4^+^CD45RA^−^CXCR5^+^CXCR3^+^CCR6^−^) (D) TFH2 (CD4^+^CD45RA^−^CXCR5^+^CXCR3^−^CCR6^−^) (E) TFH17 (CD4^+^CD45RA^−^CXCR5^+^CXCR3^−^CCR6^+^) and (F) TFH17.1 (CD4^+^CD45RA^−^CXCR5^+^CXCR3^+^CCR6^+^). Data are presented as mean ± SD. Statistical significance: ^∗^ *p* < 0.05. TFH, T follicular helper.

Next, we analyzed the distinct circulating T cell subpopulations regarding their functional properties (CD45RA, CD8, CD4, IL‐4, CXCR5, IL‐10, CD25, IL‐2, IL‐17A, CXCR3, CCR6 and IFNg) before and after 6 months of treatment using the same approach as indicated above. This analysis using surface and intracellular markers identified eight distinct clusters (A–H), each characterized by a unique expression pattern of the investigated markers (Figure 3A). Again, the composition and characteristics of T cell subsets after 6 months of anti‐IL‐4Rα treatment (Figure 3B) changed significantly. Notably, a decrease in Th2 cytokine expression (Cluster D, IL‐4) was observed, while an increase in the regulatory T cell populations (cluster F, IL‐10, CD25, CD127). Furthermore, a shift towards Th1 (cluster D, IFNg) and decreased Th17 expression (cluster F, IL‐17A) was observed.

T follicular helper (TFH) cells have been suggested to play an important role in the pathophysiology of the allergic immune response. Therefore, first we phenotypically analyzed these subpopulations in more detail by considering TFH1 (CD4^+^CD45RA^−^CXCR5^+^CXCR3^+^CCR6^−^), TFH2 (CD4^+^CD45RA^−^CXCR5^+^CXCR3^−^CCR6^−^), TFH17 (CD4^+^CD45RA^−^CXCR5^+^CXCR3^−^CCR6^+^) and TFH17.1 (CD4^+^CD45RA^−^CXCR5^+^CXCR3^+^CCR6^+^) based on surface marker expression of indicated markers. Following the identification of viable CD3^+^ T cells through flow cytometry, distinct analyses were conducted on CD3^+^CD4^+^ subset. We observed a comparable expression of TFH1 and TFH2 cells before and after 6 months of intervention in the CD4 compartment (Figure 3C,D).

Next, we assessed the frequencies of TFH17 and CD4^+^ TFH17.1 cells following IL‐4Rα blockade. A significant increase in CD4^+^ TFH17 cell frequency was observed (mean +40% from baseline, *p* < 0.05) (Figure 3E). Similarly, the CD4^+^ TFH17.1 subset exhibited a notable increase (mean +42% from baseline, *p* < 0.05) (Figure 3F).

### 4.4 Increased IL‐2 and IL‐10 and decreased IL‐4 and IL‐17 secreting T cells

**FIGURE 4:**
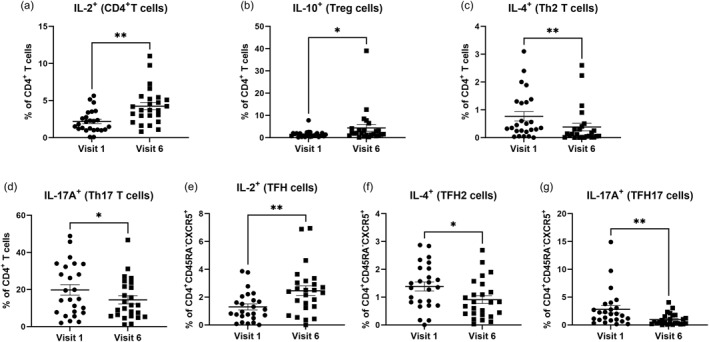
Anti‐IL‐4Rα treatment enhances the frequency of peripheral IL‐2 and IL‐10 producing T cells but reduces IL‐4 and IL‐17 producing T cells. Percentages of (A) CD4^+^ IL‐2^+^ (B) CD4^+^ IL‐10^+^ (C) CD4^+^ IL‐4^+^ (Th2 T cells) (D) CD4^+^ IL‐17A^+^ (Th17 T cells) (E) CD4^+^CD45RA^−^CXCR5^+^IL‐2^+^ (F) CD4^+^CD45RA^−^CXCR5^+^IL‐4^+^ and (G) CD4^+^CD45RA^−^CXCR5^+^IL‐17^+^ T cells in PBMCs derived from AD patients before and after 6 months of dupilumab treatment. Data are given as mean ± SD (*n* = 25). Statistical significance: ^∗^ *p* < 0.05, ^∗∗^ *p* < 0.01. Th2, T helper Type 2, Th17, T helper Type 17, TFH, T follicular helper.

Beside the phenotypic characterization of the T cell subsets, we also studied their functional capacities. Interestingly, we determined an enhanced frequency of IL‐2 producing CD4^+^ T cells (mean +0.96 fold from baseline, ^∗∗^ *p* < 0.01) (Figure 4A). The analysis of IL‐10 producing Treg cells (CD4^+^CD25^+^CD127^−^IL‐10^+^) revealed an increased frequency of IL‐10 producing Tregs (mean +1.96 fold from baseline, ^∗^ *p* < 0.05) (Figure 4B), while the frequencies of IL‐4 (mean −0.55 fold from baseline, ^∗∗^ *p* < 0.01) and IL‐17A (mean −0.27 fold from baseline, ^∗^ *p* < 0.05) producing CD4^+^ T cells were significantly decreased upon targeting IL‐4Rα (Figure 4C,D). In line with data from the CD4^+^ subset, we also observed an upregulation of IL‐2 producing CD4^+^ TFH cells (mean +0.90 fold from baseline, ^∗∗^ *p* < 0.01), and less IL‐4 (TFH2) (mean −0.34 fold from baseline, ^∗^ *p* < 0.05) and IL‐17A (Th17) (mean −0.66 fold from baseline, ^∗∗^ *p* < 0.01) CD4^+^ TFH cells (Figure 4E–G). The cytokine production of circulating total CD8^+^ T cells or the TFH‐like‐CD8^+^ cell subset (data not shown) remained unchanged.

### 4.5 IgE response and eosinophil counts

**FIGURE 5:**
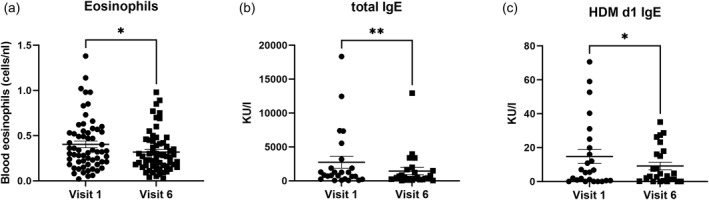
Decrease of eosinophils and IgE production upon dupilumab treatment. (A) Blood eosinophil count (*n* = 60), (B) serum total IgE, and (C) HDM *Der p1*‐specific IgE levels in atopic dermatitis patients pre‐ and post‐anti‐IL‐4Rα treatment. Data are presented as mean ± SD (*n* = 75 for clinical response, *n* = 25 for IgE levels). Statistical significance: ^∗^ *p* < 0.05, ^∗∗^ *p <* 0.005. Ig, immunoglobulin; HDM, house dust mite.

We next examined TH2 cytokine associated immune parameters such as peripheral blood eosinophils and IgE. The eosinophils were reduced after 6 months of treatment (mean −22% from baseline, ^∗^ *p* < 0.05) while other leukocytes remained stable (Figure 5A, Figure S1a–e). Next, we investigated the production of total, but also specific IgE, which are both TH2‐dependent and as IL‐4Rα is also expressed on B cells, promoting their proliferation and IgE production.^22^ In line with previous data,^23^, ^24^ we observed decreased total IgE (mean −0.47 fold from baseline, ^∗∗^ *p* < 0.01) (Figure 5B) and specific IgE (Der p1) (mean −0.40 fold from baseline, ^∗^ *p* < 0.05) (Figure 5C) while House Dust Mite (HDM) Der p2‐specific IgE (Figure S1f) showed a clear trend but did not reach statistical significance (*p =* 0.0665). By contrast, the IgE‐R network remained unchanged as neither sCD23 (*p =* 0.39, Figure S1h) nor soluble FcεR1 (*p =* 0.41, Figure S1h) were altered. Soluble CD23 promotes IgE production and cytokine release, while sFcεRI binds IgE and modulates receptor signaling. Both are Th2‐related markers and the proposed biomarkers of allergic disease activity.^21^ The absence of significant changes in their serum levels following treatment suggests that dupilumab may not have a direct or measurable effect on these specific soluble markers in the short term despite clinical improvement. These findings may reflect the complexity of immune modulation in AD as dupilumab ameliorates IL‐4 and IL‐13 signaling but may not fully interfere with other biomarkers of Th2 activity, especially those related to B cell or Fc receptor pathways.

## 5 DISCUSSION

To provide a deeper understanding of the T cell homeostasis and consecutive Th2‐dependent allergic immune responses in AD patients upon systemic IL‐4Rα targeting we analyzed peripheral T cells phenotypically and functionally. Our data show that targeting of IL‐4Rα after 6 months not only improves the clinical symptoms of AD but also results in significant systemic changes of the T cell subset clustering and their cytokine production profiles and its associated outcome parameters like total and specific IgE levels and eosinophils.

Initially, we studied the central and effector T cell memory compartment and observed a notable increase in TCM and TEM T cells during dupilumab in the memory T cell compartment. As memory T cells are responsible for long‐term immunosurveillance and a rapid immune response to antigen re‐exposure^25^ their expansion suggests that anti‐IL‐4Rα treatment may enhance the T cell dependent immunological readiness and effector functions. Previous studies including our own previous data^3^, ^26^, ^27^ have similarly reported an enhanced memory T cell compartment in AD patients. Conversely, the lower presence of TEMRA cells may suggest a reduced cytotoxic potential and tissue inflammation.^28^, ^29^ The reduction of TEMRA cells after treatment with anti‐IL4Rα shows that this biologic may be able to attenuate excessive inflammatory responses. Overall, these findings suggest that anti‐IL‐4Rα targeting promotes a systemic transition from a naive dominated to a central and effector memory T cell dominated ratio.

The detection of reduced Th2 cells which are key drivers of allergic inflammation through the production of IL‐4 and IL‐13^30^ are in alignment with previous studies.^22^, ^24^ Moreover, we unraveled an enhanced Treg activity following IL‐4Rα blockade,^3^ particularly of those Tregs producing IL‐10. The systemic increase of these cells may suggest that dupilumab promotes a balanced immune regulation and reduces systemic inflammation by enhancing the functions of the Tregs.

TFH cells are functionally heterogeneous, with subtypes including TFH1 (produces IFN‐γ and IL‐21, associated with Th1 responses), TFH2 (produces IL‐4, IL‐13 and IL‐21, promoting IgE class switching and allergic responses), TFH17 (produces IL‐17, IL‐21 and IL‐22, involved in autoimmune and inflammatory conditions), and TFH17.1 (produces IFN‐γ and IL‐17, bridging Th1 and Th17 responses).^31^, ^32^, ^33^, ^34^ TFH17 cells play an important role in the pathogenesis of autoimmune and allergic disorders by promoting B cell activation and driving chronic inflammation.^35^, ^36^, ^37^, ^38^, ^39^ In extrinsic AD (characterized by high IgE levels), TFH cells support IgE production and B cell responses, with elevated levels of circulating CXCR5^+^ PD‐1^+^ ICOS^+^ TFH‐like cells observed in children with AD and are associated with increased IL‐21 production.^40^ Dupilumab treatment in AD alters the dynamics of TFH subsets, indicating that these cells may respond to therapeutic interventions and influence treatment outcomes.^9^, ^41^ Our data highlight a modulation of circulating TFH cell subsets upon dupilumab treatment with an increase in both systemic TFH17 and TFH17.1 cells. However, it is important to note that the changes in TFH17 and TFH17.1 levels were not sustained over the long‐term treatment period and appeared to be transient.^9^ This transient increase aligns with the observed decrease in IL‐4 and IL‐17 production, suggesting a decrease in TFH2 and TFH17 activity over time. These findings indicate that while dupilumab initially shifts TFH cell dynamics, it ultimately promotes the long‐term normalization of the inflammatory profile by reducing TFH2‐ and TFH17‐mediated cytokine responses, contributing to its sustained therapeutic efficacy. The increase in TFH17 and TFH17.1 cells, which can be associated with immune regulatory functions, suggests that dupilumab may also promote a more balanced humoral response in B cells from AD patients. This finding is novel and warrants further investigation to elucidate the precise mechanisms underlying TFH cell modulation by dupilumab. The modulation of TFH cells observed in our study aligns with a broader understanding of how IL‐4Rα blockade influences B cell responses and IgE production.^42^, ^43^

On the other hand, IL‐17 producing cells play a central role in the pathophysiology of psoriasis.^44^ TFH17 cells are central to disease progression by producing IL‐17 and IL‐21, which drive keratinocyte proliferation and sustain the inflammatory environment.^45^ TFH17 cells also play a key role in memory maintenance, supporting long‐term antibody responses and contributing to chronic immune regulation.^46^

The increased IL‐2 production and attenuated IL‐4 and IL‐17A production by CD4^+^ T cells after dupilumab treatment may alter Treg cell development and function,^47^, ^48^ supporting the observed increase in Treg populations. The reduction of IL‐4 and IL‐17A positive cells, both pro‐inflammatory cytokines, indicate a shift towards a more regulatory immune milieu, which is further fostered by enhanced IL‐2. IL‐2 conjugates are currently under investigation for AD and other disorders.^49^, ^50^ These observed changes in cytokine production are consistent with a previous report of immunological shifts required to mitigate AD pathology.^3^, ^26^, ^51^

Finally, and probably as a consequence of the systemic T cell and also B cell modulation, we observed a reduction of total serum IgE levels and specific IgE against HDM following anti‐IL‐4Rα treatment. This observation aligns with previous studies that reported similar findings.^4^, ^24^, ^26^, ^52^ IgE facilitates mast cell degranulation and the release of pro‐inflammatory mediators.^53^, ^54^ Its reduction suggests that this pathogenic pathway is disrupted as well, thereby alleviating the allergic inflammation including other allergic manifestations such as allergic rhinitis or bronchial asthma. Indeed, previous and current clinical data have shown at least for asthma efficacy of dupilumab on these comorbidities. The decrease in peripheral eosinophils^55^ over a period of 6 months supports the systemic anti‐allergic capacity of dupilumab. However, the cause of the transient increase in eosinophils after treatment initiation is not exactly known, but might be a result of re‐migration of tissue located in the eosinophils into the peripheral blood.

Taken together, our data show a reduction in peripheral Th2 cells and its related functional parameters. The observed changes in TFH subsets, including TFH2, TFH17, and TFH17.1, along with their associated cytokines, suggest that dupilumab leads to an alteration of these TFH populations, contributing to the restoration of the dysregulated immune response in AD. This targeted adjustment in TFH cell dynamics may play a role in reducing the systemic allergic response. Further research is necessary to validate these findings in larger cohorts and to explore the long‐term impact of anti‐ IL‐4Rα therapy on T cell dynamics in AD.

## AUTHOR CONTRIBUTIONS

**Davender Redhu**: Methodology; writing—review and editing; formal analysis; writing—original draft. **Wojciech Francuzik**: Writing—review and editing; writing—original draft; formal analysis. **Philipp Globig**: Writing—review and editing; project administration. **Margitta Worm**: Conceptualization; funding acquisition; writing—review and editing; supervision.

## CONFLICT OF INTEREST STATEMENT

The authors declare no conflicts of interest.

## Supporting information

Supporting Information S1

Figure S1

## Data Availability

Data will be provided on a reasonable request from the corresponding author.
