# Supplementary material for: T cell immunophenotypes and IgE responses in patients with moderate‐to‐severe atopic dermatitis receiving dupilumab
Source: Clin Transl Allergy. 2025 May 8;15(5):e70062. doi: 10.1002/clt2.70062 (PMC12061530; doi:10.1002/clt2.70062)
Supplement: Supplementary file 1 — Supporting Information S1 [file CLT2-15-e70062-s001.docx]

**Supplementary Material**

**Figure Legend**

S. fig. 1 **Anti-IL4Rα treatment did not alter peripheral leukocytes except eosinophils and HDM Der p2 specific IgE in AD patients.** Blood count of (a) leukocytes and (b) lymphocytes (c) monocytes (d) basophils (e) neutrophils. Serum levels of (f) HDM Der p2 specifc IgE (g) soluble CD23 and (h) FcεR1 in AD patients pre and post anti-IL-4Rα treatment. Data were presented as mean ± SD, A-E (n=60), F (n=25) G-H (n=16) Ig, Immunoglobulin, HDM, House Dust Mite.

Table S1

| **Antibody** | **Fluorochrome** | **Supplier** | **Clone** | **Dilution** |
| --- | --- | --- | --- | --- |
| CD45RA | FITC | Biolegend | HI100 | 1:20 |
| CCR7 | PE | Biolegend | G043H7 | 1:20 |
| PD1 | PC5.5 | Biolegend | EH12.2H7 | 1:20 |
| CD27 | PC7 | Biolegend | LG. 3A10 | 1:20 |
| CD4 | APC | Biolegend | OKT4 | 1:20 |
| CD8 | A700 | Biolegend | SK1 | 1:20 |
| CD3 | APC-A750 | Biolegend | UCHT1 | 1:20 |
| CD57 | Pacific Blue | Biolegend | HNK-1 | 1:20 |
| CD25 | ECD | Beckman coulter | B1.49.9 | 1:10 |
| CD127 | BV785 | Biolegend | A019D5 | 1:20 |
| Zombie | NIR | Invitrogen | Dye | 1:200 |

Antibodies and live/dead (Zombie) dye used for phenotypic characterization of T cells are listed in Table S1

Table S2

| **Antibody** | **Fluorochrome** | **Supplier** | **Clone** | **Dilution** |
| --- | --- | --- | --- | --- |
| IFNγ | Spark PLUS UV395 | Biolegend | 4S.B3 | 1:50 |
| CD25 | BV421 | Biolegend | M-A251 | 1:100 |
| IL-2 | BV510 | Biolegend | [JES6-5H4](https://www.biolegend.com/de-at/search-results?Clone=JES6-5H4) | !.50 |
| IL-17 | BV605 | Biolegend | BL168 | 1:20 |
| CXCR3 | BV650 | Biolegend | CXCR3-173 | 1:20 |
| CCR6 | BV785 | Biolegend | G034E3 | 1:100 |
| CD45RA | FITC | Biolegend | HI100 | 1:1000 |
| CD4 | Per/CP-Cy5.5 | Biolegend | OKT4 | 1:50 |
| IL-4 | PE | Biolegend | MP4-25D2 | 1:500 |
| CXCR5 | PE-Dazzle | Biolegend | J252D4 | 1:500 |
| CD127 | PE-Cy7 | Biolegend | A019D5 | 1:500 |
| IL-10 | AF647 | Biolegend | JES3-9D7 | 1:50 |
| CD8 | ECD | Beckman coulter | SFCI21Thy2D3 | 1:10 |
| CD3 | AF700 | Biolegend | OKT3 | 1:200 |
| Zombie | NIR | Invitrogen | Dye | 1:200 |

Antibodies and live/dead (Zombie) dye used for functional characterization of T cells are listed in Table S2
