# Supplementary figures and images for: T cell immunophenotypes and IgE responses in patients with moderate‐to‐severe atopic dermatitis receiving dupilumab
Source: Clin Transl Allergy. 2025 May 8;15(5):e70062. doi: 10.1002/clt2.70062 (PMC12061530; doi:10.1002/clt2.70062)

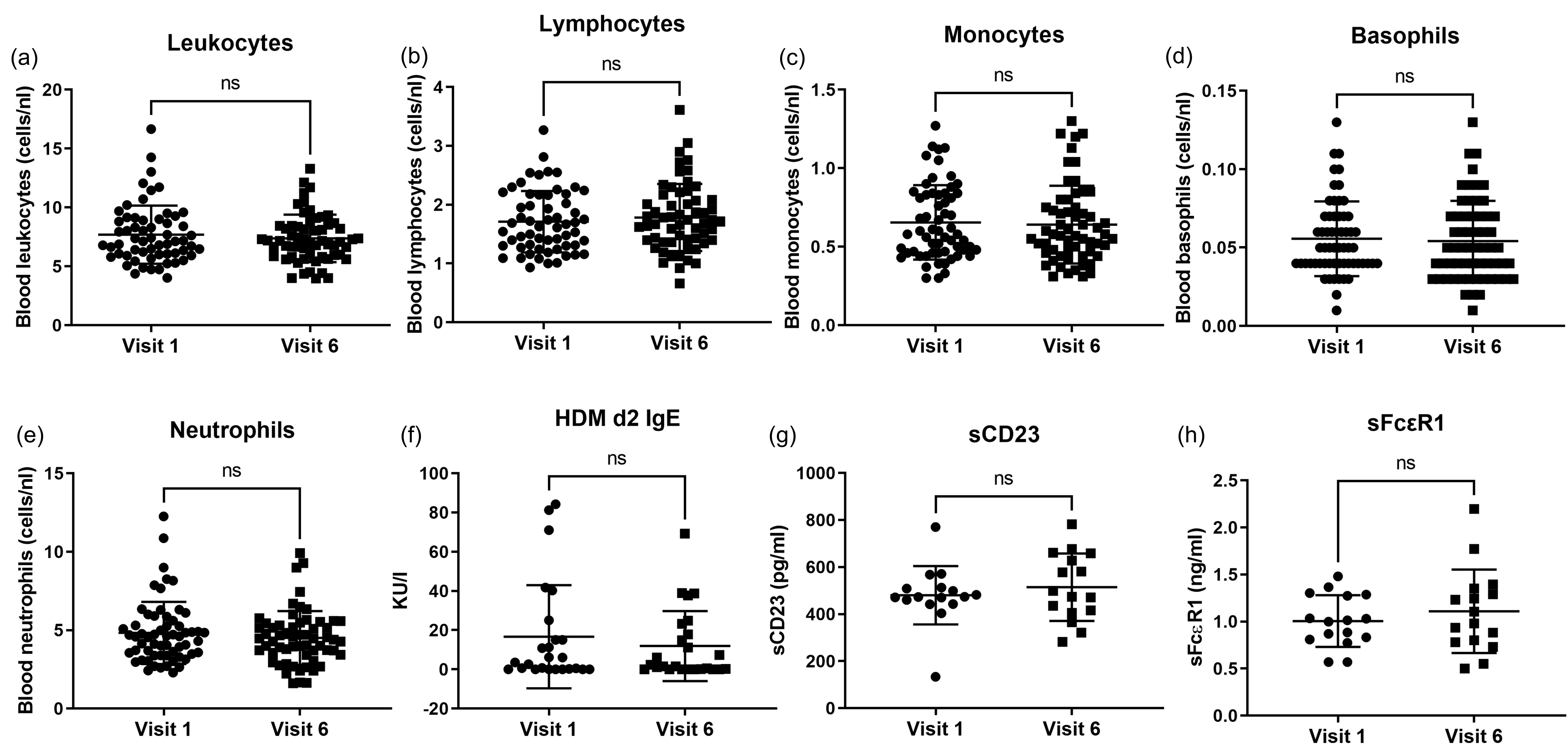

Supplement: Supplementary file 2 — Figure S1 [file CLT2-15-e70062-s002.tiff]
